# Modeling first order additive × additive epistasis improves accuracy of genomic prediction for sclerotinia stem rot resistance in canola

**DOI:** 10.1002/tpg2.20088

**Published:** 2021-02-24

**Authors:** Mark C Derbyshire, Yuphin Khentry, Anita Severn‐Ellis, Virginia Mwape, Nur Shuhadah Mohd Saad, Toby E Newman, Akeem Taiwo, Roshan Regmi, Lone Buchwaldt, Matthew Denton‐Giles, Jacqueline Batley, Lars G Kamphuis

**Affiliations:** ^1^ Centre for Crop and Disease Management, School of Molecular and Life Sciences Curtin University Perth Western Australia Australia; ^2^ School of Biological Sciences, University of Western Australia Perth Western Australia Australia; ^3^ Agriculture and Agri‐Food Saskatoon Saskatchewan Canada; ^4^ Corteva Agriscience New Plymouth Taranaki New Zealand

## Abstract

The fungus *Sclerotinia sclerotiorum* infects hundreds of plant species including many crops. Resistance to this pathogen in canola (*Brassica napus* L. subsp. *napus*) is controlled by numerous quantitative trait loci (QTL). For such polygenic traits, genomic prediction may be useful for breeding as it can capture many QTL at once while also considering nonadditive genetic effects. Here, we test application of common regression models to genomic prediction of *S. sclerotiorum* resistance in canola in a diverse panel of 218 plants genotyped at 24,634 loci. Disease resistance was scored by infection with an aggressive isolate and monitoring over 3 wk. We found that including first‐order additive × additive epistasis in linear mixed models (LMMs) improved accuracy of breeding value estimation between 3 and 40%, depending on method of assessment, and correlation between phenotypes and predicted total genetic values by 14%. Bayesian models performed similarly to or worse than genomic relationship matrix‐based models for estimating breeding values or overall phenotypes from genetic values. Bayesian ridge regression, which is most similar to the genomic relationship matrix‐based approach in the amount of shrinkage it applies to marker effects, was the most accurate of this family of models. This confirms several studies indicating the highly polygenic nature of sclerotinia stem rot resistance. Overall, our results highlight the use of simple epistasis terms for prediction of breeding values and total genetic values for a complex disease resistance phenotype in canola.

## Core Ideas

- LMMs predicted sclerotinia stem rot resistance in canola.
- Incorporating epistasis improved accuracy and predictive ability.
- Bayesian models performed similarly to or worse than simple LMMs.
- Accounting for epistasis may be useful for applying genomic selection to this trait.

**Abbreviations**
AGG: Australian Grains Genebank
ASSYST: Associative expression and systems analysis of complex traits in oilseed rape/canola
AUDPC: area under the disease progress curve
BLUP: best linear unbiased predictor
DPI: days post inoculation
G‐BLUP: genomic best linear unbiased predictor
GEBV: genomic estimated breeding value
*h*
: square root of narrow‐sense heritability
*H* ^2^
: broad‐sense heritability
*h* ^2^
: narrow‐sense heritability
LMM: linear mixed model
MCMC: Markov chain Monte Carlo
PSCL: proportion of soft or collapsed lesions
QTL: quantitative trait loci
RR‐BLUP: ridge regression best linear unbiased predictor
SNP: single nucleotide polymorphism.

## 1 INTRODUCTION

The fungus *Sclerotinia sclerotiorum* infects several hundred plant species in diverse dicotyledonous families (Boland & Hall, 1994). Many of its hosts are important crops, in which it may substantially reduce yield in conditions conducive to infection (Ficke et al., 2018). One of the most economically important oilseed crops infected by *S. sclerotiorum* is canola (*Brassica napus* L. subsp. *napus*), in which the disease is often referred to as sclerotinia stem rot (Derbyshire & Denton‐Giles, 2016).

A number of studies have reported varying levels of resistance to *S. sclerotiorum* in *B. napus* (Denton‐Giles et al., 2018; Gyawali et al., 2016; Uloth et al., 2015; Wu et al., 2019). The genetic loci controlling resistance to *S. scerotiorum* in *B. napus* have also been investigated using genome‐wide association and biparental mapping (Behla et al., 2017; Gyawali et al., 2016; Wu et al., 2013, 2016, 2019; Zhao et al., 2006). Using quantitative trait locus (QTL) mapping in a doubled‐haploid population, Wu et al. (2013) identified a strong candidate causal variant for *S. sclerotiorum* resistance residing within a *B. napus* gene homologous to the Arabidopsis [*Arabidopsis thaliana* (L.) Heynh.] gene *Indole Glucosinolate Methyl‐transferase 5* (*IGMT5*). Indole glucosinolates are plant secondary metabolites with varied roles in innate immunity to pathogens (Pfalz et al., 2016). This could mean that variations in this aspect of innate immunity in *B. napus* contribute substantially to resistance to *S. sclerotiorum*.

Also using QTL mapping in a doubled‐haploid population, Wu et al. (2019) showed that QTL conferring resistance to *S. sclerotiorum* contributed to flowering time. This echoes several studies detailing genetic factors shared between disease resistance and flowering response pathways in plants. For example, the salicylic acid regulator *WIN3* controls broad‐spectrum resistance to disease in Arabidopsis while also moderating the expression of flowering regulatory genes (Banday & Nandi, 2015; Wang et al., 2011).

Despite the growing body of knowledge on the genetic basis of *S. sclerotiorum* resistance in *B. napus*, there have been limited commercial introductions of canola cultivars with sclerotinia stem rot resistance. So far, most resistant cultivars have been released in Canada, where they are reported to develop <60% of the disease incidence found in susceptible check varieties (World Patent Organization number WO 2006/135717 A1). The sclerotinia stem rot resistance trait they carry has been marketed as Protector by DuPont Pioneer and appears to be partly based on QTL identified by Zhao et al. (2006) and Zhao and Meng (2003).

Quantitative trait loci identified through genome‐wide association mapping so far individually explain a maximum of 25% of phenotypic variance (Gyawali et al., 2016), indicating that there is a significant amount of untapped genetic potential in *B. napus* for sclerotinia stem rot resistance breeding. To optimally exploit genetic potential for this trait using genome‐assisted breeding, models incorporating multiple QTL are needed. Genomic prediction is a technique commonly used for improving polygenic traits such as sclerotinia stem rot resistance. This technique uses an array of genetic markers and phenotypic data to develop a statistical model that predicts breeding values or total genetic values in individuals based on their genotypes.

A standard statistical framework for genomic selection is the linear mixed model (LMM). Under this framework, a random effect with variance conditioned on allele sharing between individuals is used to derive a best linear unbiased predictor (BLUP) for the trait of interest (Henderson, 1973). Before genotype data were available, the variance–covariance matrix used to estimate BLUPs was a relationship matrix based on a pedigree, which describes the fraction of allele sharing between individuals based on their relationships to one another. In genomic prediction, replacement of the pedigree relationship matrix with a genomic relationship matrix that describes the actual, or realized, relationships between individuals may improve accuracy by accounting for Mendelian sampling of alleles, an approach known as genomic BLUP (G‐BLUP) (VanRaden, 2008).

Equivalent to G‐BLUP, with a realized genomic relationship matrix, is ridge regression BLUP (RR‐BLUP), which models marker effects as independent and normally distributed (Meuwissen et al., 2001). The marker effects framework lends itself to more complex hierarchical Bayesian regression models. These models may use different priors that cause different levels of effect size shrinkage with different proportions of zero effect markers (Meuwissen et al., 2001; Habier et al., 2011; Erbe et al., 2012). For some traits, this approach may be more accurate than RR‐BLUP or G‐BLUP, as it allows for a more realistic representation of the trait genetic architecture (Meuwissen et al., 2001; Resende et al., 2012). These models may be particularly accurate for moderately to highly heritable traits controlled by a relatively small number of QTL (Resende et al., 2012; Barili et al., 2018; Zhang et al., 2019).

A major aim of genomic prediction in the early stages of breeding programs is to capture additive genetic variance to estimate breeding values, known as genomic estimated breeding values (GEBVs) (Abera Desta & Ortiz, 2014). Additive genetic variation is likely to make a stable contribution to phenotype between generations, whereas nonadditive variation that causes changes in dominance and epistasis effects is transient and will likely have different impacts in different individuals. However, additive genetic variance may be confounded by epistasis and dominance effects for some traits in some populations as the three effects are not usually orthogonal (Hill et al., 2008; Muñoz et al., 2014). If this is the case, GEBVs can inadvertently incorporate transient effects that make them less stable and accurate (Varona et al., 2018).

In the G‐BLUP context, one approach to improve genetic variance partitioning is the use of transformations or reformulations of the realized genomic relationship matrix to capture different genetic effects. For example, Muñoz et al. (2014)⁠ used the Hadamard product of the additive genomic relationship matrix to model first‐order epistasis and a genomic relationship matrix based on heterozygous loci to model dominance effects. By distributing genetic variance estimates between additive and nonadditive terms, it was shown that the accuracy and stability of GEBVs might be significantly improved. Modeling nonadditive genetic effects at later stages of the breeding program when selecting cultivars may also be advantageous, as it can assist with prediction of total genetic value of candidates for commercial release (Crossa et al., 2017).

Several studies have tested the accuracy of genomic prediction for disease traits in crops including canola (Poland & Rutkoski, 2016). Fikere et al. (2018) tested the accuracy of genomic prediction for blackleg resistance in a *B. napus* population, and found that within environments, predictive ability ranged from moderate, with a Pearson's ρ of .35, to high, with a ρ of .74, when comparing GEBVs with trait scores through cross‐validation. Prediction between environments in this study was lower with a ρ of .28–.58, indicating potential genotype × environment interactions.

Since sclerotinia stem rot is a trait that appears to be controlled by numerous fairly minor‐effect QTL in natural populations, it is highly suited to genomic prediction for breeding purposes. However, no studies have tested the suitability of different genomic selection models to this trait. Therefore we aimed to assess (a) whether including first‐order additive × additive epistasis effects in a G‐BLUP model improved model fit and stability of breeding value and total genetic value estimation; (b) whether different Bayesian priors had an impact on breeding value and phenotype prediction accuracy; and (c) whether phenotypic measurements were stable between different typical plant screening environments for this trait using different pathogen isolates. This preliminary study on a diverse panel of spring *B. napus* plants provides a starting point for further investigations into the use of genomic selection for sclerotinia stem rot resistance breeding.

**Core Ideas**

- LMMs predicted sclerotinia stem rot resistance in canola.
- Incorporating epistasis improved accuracy and predictive ability.
- Bayesian models performed similarly to or worse than simple LMMs.
- Accounting for epistasis may be useful for applying genomic selection to this trait.

## 2 MATERIALS AND METHODS

### 2.1 Screening of *Brassica napus* lines for sclerotinia stem rot susceptibility

A total of 218 *B. napus* lines were obtained from Professor Rod Snowdon (University of Geissen), the Australian Grains Genebank (AGG), and Gyawali et al. (2016) (Supplemental Table S1). The lines obtained from Professor Snowdon were first described in Bus et al. (2011) and were collected on the European Research Area Network project ‘Associative expression and systems analysis of complex traits in oilseed rape/canola’ (ASSYST). Three screens were performed: in 2016, 2017, and 2019. The 2016 and 2017 screens were performed on 99 of the ASSYST varieties, whereas the 2019 screen was performed on the same 99 varieties, an additional 115 varieties from AGG, and the four sclerotinia stem rot resistant accessions PAK93, PAK54, DC21, and K22 from Gyawali et al. (2016).

During the 2016 and 2017 trials, *B. napus* plants were grown in a hoop house in 4‐L pots containing compost (UWA plant biology mix, available from Richgro, Australia) with drip irrigation every 2 d. They were fertilized with Nitrophoska Perfekt prior to bolting.

When all plants had reached at least 30% flowering, they were inoculated with 3 mm potato dextrose agar plugs containing the *S. sclerotiorum* isolate CU11.19 (Derbyshire et al., 2019). Plants were inoculated approximately halfway between the base and the oldest flowering branch by wrapping plugs to the stems using Parafilm with fungal mycelium facing the stem. Downstream analyses were performed using measurements of lesion length at 21 d post inoculation (DPI). Pots were arranged in a completely randomized design on benches throughout the hoop house.

During the 2019 screen, varieties were grown in a temperature‐controlled glasshouse in 5‐L pots containing potting soil (UWA plant biology mix) and irrigated twice daily with drip irrigation for 1–2 min. Each pot received 2 g of fertilizer comprising 7.3% total nitrogen, 27.8% potassium, 11% phosphorus, and 2.7% sulfur, and 0.21% iron, 0.1% manganese, 0.01% copper, 0.06% zinc, 0.08% boron, and 0.08% molybdenum as trace elements (Diamond Red, Campbell's Fertilizers Australasia, Australia). The fertilizer was dissolved in 125 ml of water and applied individually to each pot every 14 d. Positions of pots within the glasshouse were completely randomized. Temperature settings were maintained between 12 and 24 °C.

When a plant reached 30–50% flowering, it was tagged. Each week, all tagged plants were inoculated with 5 mm potato dextrose agar plugs containing *S. sclerotiorum* isolate CU8.24 (Derbyshire et al., 2019). Plants were inoculated approximately halfway between their bases and lowest flowering branches by wrapping the agar plugs onto the stems with Parafilm with fungal mycelium facing them. Plants were subsequently scored at 7, 14, and 21 DPI by measuring lesion lengths. Downstream analyses were conducted using area under the disease progress curve (AUDPC) measurements based on scores at these three time points. At 21 DPI, lesions were also assessed to determine whether they were firm (including black, aborted lesions), soft, or collapsed. A score of the proportion of soft or collapsed lesions (PSCL) among replicates was recorded for each line.

For all data sets, a single plant in a single pot was considered a single replicate. In 2016 and 2017, varieties were replicated four and eight times, respectively, in a hoop house. In 2019, varieties were replicated six times in a temperature‐controlled glasshouse. The three data sets are in Supplementary File S1.

### 2.2 Statistical adjustment of susceptibility scores for the 2019 data set

During the 2019 screen, there was some variability from week to week in relative humidity and the culture of *S. sclerotiorum* isolate CU8.24 used. This was a compromise that was deemed necessary to control for the potential confounding factor of plant developmental age at time of inoculation. To deal with the effects of this variability on disease scores, a LMM was fit to data treating inoculation date as a fixed factorial effect and variety as a random factorial effect. Best linear unbiased predictors were retained from this model and used as the dependent variable in genomic selection models. This model was also used to estimate broad‐sense heritability (*H* ^2^), the amount of variance explained by intervarietal differences, without genotypic data. Herein, the model without genetic variance matrices is referred to as the stage‐one model and the models with them are referred to as stage‐two models.

### 2.3 SNP genotype data for the canola population

All single nucleotide polymorphism (SNP) data sources are provided in Supplemental Table S1. A merged data set containing all SNPs for all varieties used in this study is provided in Supplemental Table S2. Genotype data for the 99 ASSYST lines were obtained from Professor Rod Snowdon (Bus et al., 2011). The genotypes of 10 varieties were obtained from the supplementary material of Mason et al. (2015), and a further three from Mason et al. (2018). Illumina reads from whole‐genome sequences of 18 varieties were obtained from the National Centre for Biotechnology Information Sequence Read Archive; 16 of these data sets were first described in Malmberg et al. (2018) and two in Wang et al. (2018). A further whole‐genome sequence data set, described in Lu et al. (2019), was downloaded from the National Genomics Data Center (https://bigd.big.ac.cn/) where it is stored under accession number CRR046026. An additional 87 varieties were genotyped in this study using the *B. napus* Illumina Infinium 60K SNP array (Clarke et al., 2016). A further 105 ASSYST winter varieties (their publicly available SNP genotypes are in Supplemental Table S3) were also considered in analyses of population structure.

For 19 varieties, whole‐genome sequence reads were mapped to the *B. napus* reference genome (Chalhoub et al., 2014) using Bowtie version 2.3.3.1 (Langmead & Salzberg, 2012) with default parameters. Reads mapping to multiple locations with equal confidence were removed using samtools version 1.6 (Li & Durbin, 2009) with the flag ‘‐q 5’ supplied to the function ‘view’. Variants were called using the program freebayes version 1.3.0‐1 (available at the time of writing at https://github.com/ekg/freebayes). As in Fikere et al. (2018), variants were filtered to remove those with a depth of <5.

All genotype data were filtered using the R package snpReady version 0.9.6 to remove any with a call rate <0.5 and a minor allele frequency <0.01. Missing SNPs were imputed using the Wright method with snpReady. The SNPs that were >40% heterozygous were also removed using R. After this filtering step, 24,634 SNPs remained. Hierarchical clustering was performed using the R package heatmap3 version 1.1.7 with the distance function ‘dist’ using the McQuitty algorithm. A principal component analysis was performed on the additive relationship matrix (detailed in a later section) using the R function ‘princomp’.

### 2.4 Genomic relationship matrix based LMMs with additive and first‐order additive × additive epistatic variance components

All LMMs were fit using the R package sommer version 4.0.9 (Covarrubias‐Pazaran, 2016). The genomic relationship matrices considered were the additive matrix proposed by Endelman (2011) and the epistatic matrix proposed by Muñoz et al. (2014). The full model was fit as a regression of varietal BLUPs onto the relationship matrices as follows:

y=μ+a+e+ε

where μ is the mean score for all lines. Different varietal random effects were estimated with their covariances conditioned on either of the different genomic relationship matrices. The effect *a* was conditioned on the additive genomic relationship matrix of Endelman (2011). Epistatic effects, *e*, were conditioned on the Hadamard product of the additive matrix. The Hadamard product of genomic relationship matrices has been shown to capture variance from first order epistatic effects by Muñoz et al. (2014). Random effect covariances were thus assumed to be distributed around the mean as $N(0,G\sigma_{\mu }^{2})$, where **G** represents the relationship matrix or its Hadamard product and $\sigma_{\mu }^{2}$ is the random varietal effect variance to be estimated for that variance component. The error term, ε, was considered normally distributed with mean zero and variance estimated from the data. Dominance was not modeled, as our data set consisted of inbred lines for which genotypes at heterozygous loci are unreliable.

Narrow‐sense heritability (*h* ^2^) from these models was assessed by dividing the variance estimate for the additive relationship matrix by the total variance estimate. For the additive‐only model, this was the additive variance divided by the sum of the additive variance and the error. For the additive and epistasis model, this was the additive variance divided by the sum of the additive variance, epistasis variance, and the error variance. Broad‐sense heritability was assessed for the additive and epistasis model by dividing the sum of the variances contributed by the two genetic components by the total variance including error.

An initial assessment of the relative contributions of additive and epistasis components to model fit was performed by assessing Akaike information criterion (Akaike, 1974) values. The model based on only the additive relationship matrix was used as a baseline to determine the improvement in fit from addition of the epistasis component.

Assessments of model accuracy and predictive ability were performed using 10 replications of 11‐fold cross‐validation (10 sets of 20 testing varieties and one set of 18). Predictive ability was defined as the correlation (Pearson's ρ) between model predictions and phenotypes. This was measured for predictions based on additive variance (GEBVs) for both models and total genetic variance (the sum of BLUPs from additive and epistatic effects) for the additive and epistasis model.

Accuracy was defined as the correlation between GEBVs from cross‐validation and an estimate of true breeding values. Although accuracy cannot be empirically assessed as the true breeding values for this trait are not known, it can be estimated by dividing the correlation (Pearson's ρ) between GEBVs and phenotypes by the square root of *h* ^2^ ($\sqrt{h^{2}}$or just *h*) (Ould Estaghvirou et al., 2013). Thus, to estimate accuracy of the models, we divided median Pearson's ρ between GEBVs and phenotypes from all cross‐validations by *h*. Values of *h* were derived from the same model fit to the full data set.

As an alternative assessment of prediction accuracy, we used the approach of Muñoz et al. (2014). We recorded GEBVs for all cross‐validation partitions and compared these with GEBVs for the same varieties estimated from the whole data set. This approach assesses the impact of individual phenotype on breeding value estimation and can give a general idea of the stability of GEBVs from a predictive model.

### 2.5 Assessment of different priors in Bayesian models

Bayesian genomic prediction models were fit using the R package BGLR version 1.0.8 (Pérez and de los Campos, 2014). These models took the following form [repeated from Pérez and de los Campos (2014)]:

y=η+ε

where η is a term describing the marker genotypes at all loci and ε is the model error. The coefficient η has the following form:

$$\eta \;=\;\mu +\sum_{j}^{J}X_{j}\beta_{j}$$

where μ is the intercept; **X** *_j_* are design matrices with individuals in rows and markers, coded as 0 and 2 for homozygotes and 1 for heterozygotes, in columns; **β** *_j_* are vectors of coefficients linking marker genotype to y. Model parameters were estimated using Markov chain Monte Carlo (MCMC), assuming one of five different priors for marker effects, Bayes A, Bayes B, Bayes C, double exponential (Bayesian LASSO) and Gaussian (Bayesian ridge regression). The MCMC chains were run for 5,000 iterations with a 1,000‐iteration burn‐in.

Predictive ability and accuracy for these models were assessed in the same way as for the genomic relationship matrix‐based LMMs previously described. Cross‐validations used the same data partitions as for these models and the additive‐only G‐BLUP model was used as a baseline to compare improvement in predictive ability and accuracy under different priors. To estimate *h* ^2^, we took the average proportion of variance ascribed to the regression of phenotypes on markers at each MCMC iteration not discarded as burn‐in (de los Campos et al., 2015).

### 2.6 Assessment of stability of phenotypic scores between plant screening environments

Most studies have assessed sclerotinia stem rot resistance similarly to us (Wu et al., 2013, 2016; Gyawali et al., 2016). These screens often use different isolates and may take place in controlled, semi‐controlled, or uncontrolled environments. We used previous screening data for 99 of the varieties tested in 2019 to assess the correlation (Pearson's ρ) in levels of resistance between different experiments performed in different conditions with a different isolate.

## 3 RESULTS AND DISCUSSION

To assess levels of resistance to *S. sclerotiorum* in canola germplasm, we screened 99 *B. napus* lines in 2016 and 2017. To assess the use of common genomic prediction models for breeding sclerotinia resistance into canola, we expanded this initial set to 218 lines in 2019. We screened this larger set with a different pathogen isolate and inoculated plants at the same flowering stage.

**FIGURE 1:**
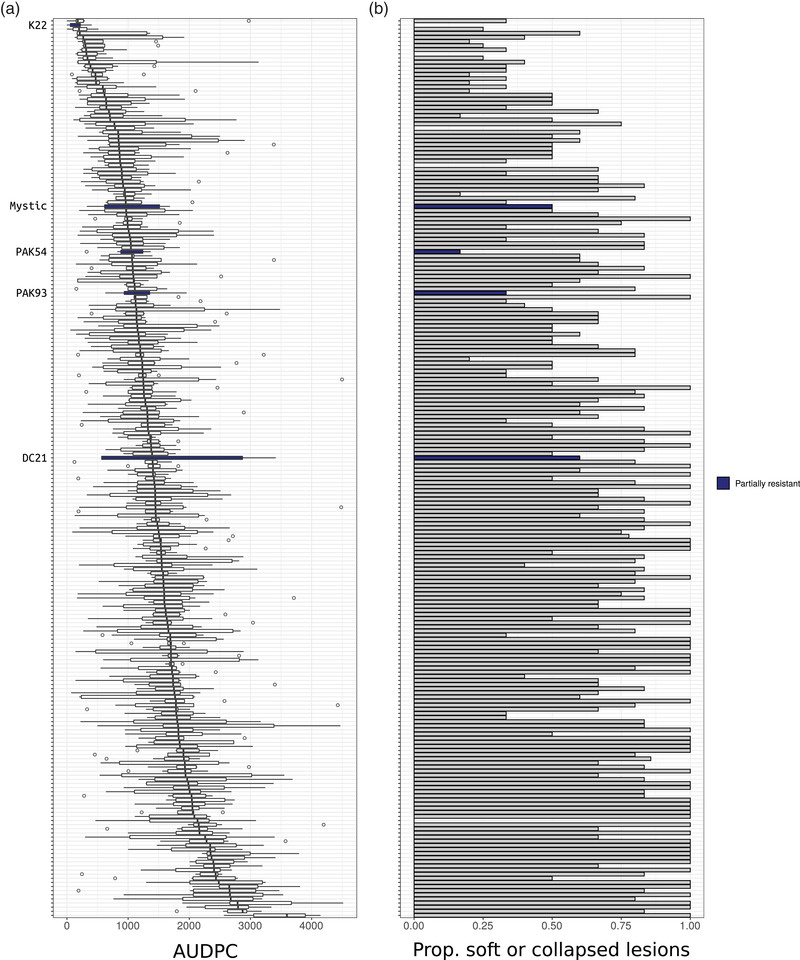
Assessment of the range of sclerotinia stem rot resistance levels in our experimental population of *Brassica napus*. (a) Area under the disease progress curve (AUDPC) (*x* axis), plotted for each variety ID (*y* axis). Black vertical bars represent median AUDPC, boxes and whiskers represent interquartile range and points represent outliers. The blue‐shaded boxes are varieties that were previously reported to have resistance to sclerotinia stem rot. (b) The same as for (A) but reporting the overall proportion of soft or collapsed lesions on the *x* axis

There was a large spread in median AUDPC scores among the varieties in 2019 (Figure 1a). In our data set, we included five accessions that were previously described as having resistance to sclerotinia stem rot: PAK93, PAK54, K22, DC21, and ‘Mystic’ (variety IDs 101, 102, 103, 104, and 108, respectively). All of these accessions, except DC21, ranked in the top 50% for resistance to SSR. Accession DC21 ranked 121 out of 218 (45th percentile), which is somewhat at odds with the literature (Denton‐Giles et al., 2018).

Previous studies on PAK93, PAK54, K22, and DC21 used a further metric to characterize disease susceptibility, that is, the PSCL (Gyawali et al., 2016; Denton‐Giles et al., 2018). This is a simple metric that records the proportion of replicate plants for each variety that had lesions soft to the touch or that had collapsed. In our data set, we found that there was a correlation between PSCL and adjusted AUDPC scores of *ρ* = .64. Moreover, plants with soft or collapsed lesions exhibited significantly higher adjusted AUDPC scores than plants without (*t* = 26.716, df = 937.46, *P* < 2.2 × 10^−16^). The resistant varieties we tested tended to score well for both AUDPC and PSCL; accession K22 (ID 103) had zero PSCL and the second lowest AUDPC (Figure 1).

The broad agreement of our data set with previous data sets suggests that resistance to *S. sclerotiorum* in *B. napus* is not strongly contingent on the pathogen isolate. Gyawali et al. (2016) used an aggressive Canadian isolate, which is very genetically distinct from the Australian isolate CU8.24 (Derbyshire et al., 2019). This is promising for resistance breeding, as it suggests that quantitative resistance to this disease is likely to be reasonably consistent across pathogen populations. However, the discrepancies we did observe, especially for accession DC21, show that that testing against multiple pathogen isolates is still desirable.

The *B. napus* lines we used were all described in the literature as either spring or semi‐winter or they were of unknown heredity (information was not available from AGG or not found online). We tried to select mostly spring varieties, as they may be most relevant to Australian breeding programs. To assess population structure, we clustered varieties based on their genotypes and performed principal component analysis. For these tests, we included an arbitrary panel of 105 additional varieties from the ASSYST collection described as winter, as genotypic differences between winter and spring lines form the main axis of genetic variation in canola populations (Lu et al., 2019).

**FIGURE 2:**
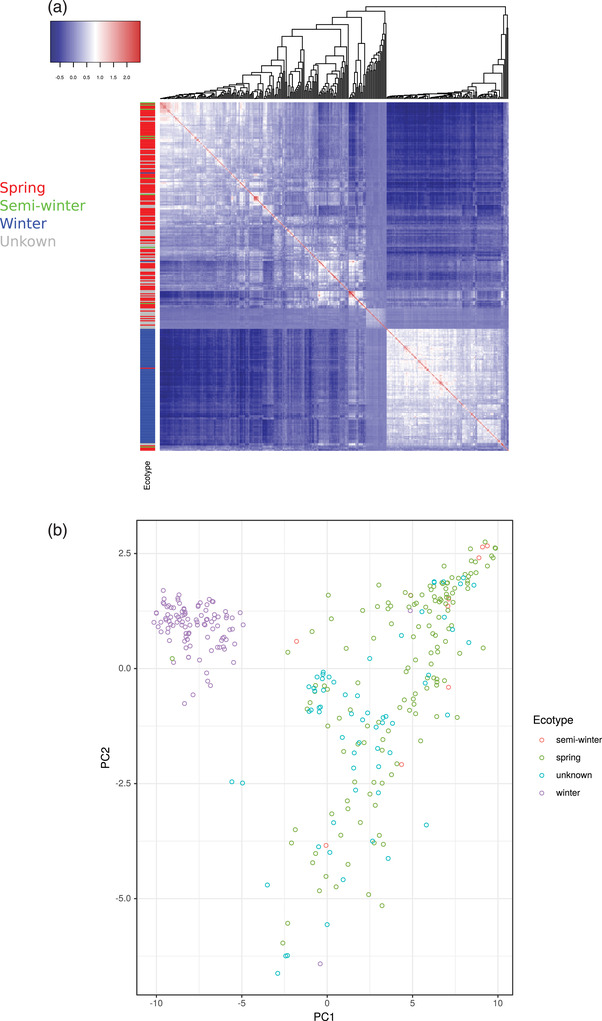
Population structure in the panel of *Brassica napus* lines assessed in this study. (a) A heatmap showing additive genetic relatedness between varieties, including an arbitrary set of 105 winter lines not phenotyped in this study. Two broad genetic clusters formed, with most of the 218 varieties included in this study falling within one cluster. Varietal descriptions from the literature of spring, semi‐winter, unknown, or winter (row colors) broadly corresponded with clustering. However, semi‐winter varieties did not group away from spring varieties and there were some varieties labeled as spring that were closer to the winter varieties, notably the cultivar Odin. (b) A principal component analysis plot of the first two principal components estimated from the same additive genomic relationship matrix as (a). Two broad clusters form along principal component 1, corresponding to winter varieties on the left and the rest of the varieties on the right

We confirmed that this was the case in our data set. Most lines fell into one of two distinct clusters (Figure 2a) that included spring, semi‐winter and unknown lines, or winter varieties. We found that the same was true for the principal component analysis—the main axis of variation was between spring, unknown and semi‐winter, and winter lines (Figure 2b). There were some exceptions to this clustering. For example, the cultivar Odin, which we found described as a spring variety in some old field reports, grouped with the winter lines; there were also a number of lines that shared heredity with both main clusters. We suspect that these lines are actually spring types with a winter background and that the rest that did not group with the winter lines are purely spring despite their descriptions. Based on the overall lack of strong subdivision within the 218 varieties, that is, the vast majority were spring, we decided to use all of them for genomic prediction testing.

Before performing genomic prediction, we assessed *H* ^2^ of AUDPC without considering any marker data. The model used to estimate this statistic was simply the fixed effect of inoculation week and the random effect of variety ID, for which variance was estimated from the data and not structured in any way. Broad‐sense heritability from this model, the stage‐one model, was .31 (SE ±.032). Thus, about one‐third of variance was caused by intervarietal differences.

The relatively large amount of residual environmental variance in this model may have been partly caused by the experimental design. There were minor fluctuations in relative humidity and temperature throughout the course of the glasshouse screen. However, we were not able to block measurements with respect to inoculation week as this was contingent on the plants reaching 50% flowering, and the date at which this was to occur was not known *a priori*. Ideally, in a plant breeding context, plants would be screened under the environment in which they are to be cultivated. However, this may be difficult for sclerotinia stem rot resistance because of the very sporadic nature of the disease (Ficke et al., 2018). Since a controlled screen is an abstraction from such a field environment anyway, it may be better in this case to attempt to mitigate as much environmental variability as possible by using a controlled environment instead of a glasshouse in future.

We then considered genetic variance from two LMMs, a model fitting a random effect with variance conditioned on an additive genomic relationship matrix and a model with an additional epistasis matrix (the Hadamard product of the additive matrix), the stage‐two models. For these models, BLUPs estimated from the stage‐one model (with inoculation week as a fixed effect) were used as the dependent variable. An overview of all models fit in this study is in Table 1. In the additive‐only model, *h* ^2^ was similar to *H* ^2^ from the stage‐one model at .30 (SE ±.11). In the additive and epistasis model, *h* ^2^ (the additive variance component) was reduced to .12 (±.12) and the epistasis component was .2 (±.12). Broad‐sense heritability (the sum of additive and epistasis components divided by the total variance) from the additive and epistasis model was .33 (±.13). This model showed a marginal improvement in fit over the additive‐only model, as its Akaike information criterion was 189, compared with 193. These data together suggest that epistasis contributes to phenotypic expression of sclerotinia stem rot resistance as measured in a glasshouse environment with an aggressive pathogen isolate in a diverse *B. napus* population.

**TABLE 1:** Overview of models fit in this study

Model^a^	AIC or DIC^b^	Narrow‐sense heritability (*h* ^2^)	Broad‐sense heritability (*H* ^2^)
LMM	1,038.55	–	.31 ± .03
**A** LMM	1,92.9	.3 ± .11	–
**A** + AA LMM	189	.13 ± .12	.33 ± .13
Bayes A	3,106.834	.39	–
Bayes B	3,149.43	.44	–
Bayes C	3,136.306	.45	–
LASSO	3,122.349	.52	–
BRR	3,156.791	.47	–

^a^ LMM, linear mixed model; **A**, additive genomic relationship matrix; AA, additive × additive epistasis (Hadamard product of **A**).

^b^ AIC, Akaike information criterion values between the nongenetic and genetic LMMs are not comparable as the nongenetic LMM was fit to replicated data with inoculation date as a fixed effect. DIC, deviance information criterion is reported for Bayesian models.

**FIGURE 3:**
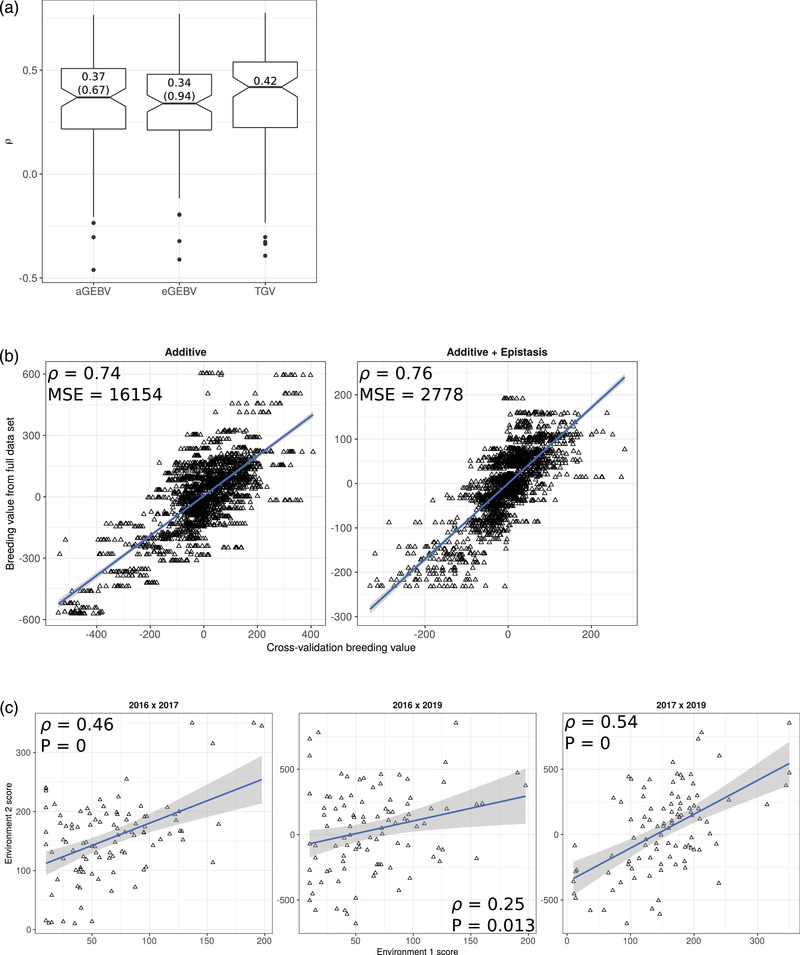
Predictive ability and accuracy of stage two models and interenvironment correlations between phenotypes. (a) Boxplot showing the results of cross‐validation for correlation between genomic estimated breeding values (GEBVs) from the additive only model (aGEBVs, left), GEBVs from the additive and epistasis model (eGEBVs, middle), or total genetic values (TGVs, right) and phenotypes of models not in the training set. Numbers above bars are median Pearson's *ρ* from cross‐validations; numbers in brackets are estimates of prediction accuracy based on division of median *ρ* × *h*. Boxes represent second and third quartiles and lines represent interquartile range; points represent outliers. (b) Plots of GEBVs from individuals in all cross‐validations on the *x* axis with GEBVs from the same individuals based on a model fit to the full data set on the *y* axis. Blue lines are a least squares regressions of *y* onto *x*; *ρ* *=* Pearson's *ρ*, MSE = mean squared error. (c) Correlations between disease resistance scores in different environments. Left is correlation between 2016 and 2017 lesion lengths; middle is 2016 lesion lengths and 2019 area under the disease progress curve (AUDPC) scores; right is 2017 lesion lengths and 2019 AUDPC scores. Blue lines are least squares regressions of *y* onto *x*; numbers show Pearson's *ρ* and associated *P* value

Next, we assessed predictive ability and accuracy of the stage‐two models. Considering the median *ρ* from 10 replicates of 11‐fold cross‐validation, we found that they had slightly different predictive abilities. The GEBVs based on the additive‐only model had a median correlation with phenotypes of .37. The GEBVs based on the additive component of the additive and epistasis model had a median correlation with phenotypes of .34. The highest median correlation, which was .42, was between total genetic values (the sum of additive and epistasis BLUPs) from the full model and phenotypic scores; this is a 14% improvement in predictive ability (Figure 3a).

One way to approximately estimate prediction accuracy (i.e. the correlation between predicted breeding values and true breeding values) is to divide the correlation between phenotypes and breeding values estimated through cross‐validation by the square root of *h* ^2^ (*h*). As the true heritability is not known, the choice of model used to identify *h* is problematic (Ould Estaghvirou et al., 2013). In our study, we chose to estimate heritability for this purpose based on the whole data set using the same model fit to the cross‐validation data set. Thus, for the additive model, *h* was .56 (square root of .31) and for the additive and epistasis model, *h* was .35 (square root of .12). This suggested that predictive accuracy was increased from .67 in the additive‐only model to .94 (∼40% increase) in the additive and epistasis model.

Stability of breeding value predictions may also be assessed through the correlation between cross‐validation breeding value estimates and breeding values derived from the full data set (Muñoz et al., 2014). Using this approach, we found that correlation between breeding value estimates from training data and from the full data increased by ∼3% from .74 to .76 going from the additive to additive and epistasis model. In addition to the slight increase in prediction accuracy when including the extra parameter, mean squared error of prediction was reduced substantially from 16,154 to 2,778 (Figure 3b).

These evaluations of prediction accuracy suggest that, on the whole, partitioning variance into additive and epistasis components using different genomic relationship matrices improves stability of breeding value estimation for sclerotinia stem rot resistance. In addition, total genetic values from these models were better correlated with phenotypes. Using nonadditive genomic relationship matrices, Liu et al. (2019) also found an improvement in prediction accuracy for grain yield per plant but not for plant height in maize (*Zea mays* L.). This is very much in accordance with the amount of variance captured by the different realizations of the genomic relationship matrix in their study. For instance, dominance and epistatic matrices captured <5% of the variance for plant height in this study, whereas the dominance matrix captured ∼17% and the epistatic matrix <5% for grain yield. Accordingly, the dominance relationship matrix gave the biggest improvements in model fit and prediction accuracy. In our study, the epistatic matrix captured ∼60% of the genetic variance, whereas, the additive genomic relationship matrix captured only 36%. The improvement in model prediction accuracy and predictive ability over an additive model in our study thus accords with the significant amount of phenotypic variance explained by the epistatic genomic relationship matrix.

Using previous data sets, we also assessed how correlated disease resistance was between different environments and pathogen isolates. Data sets from 2016 and 2017 were moderately correlated, with a *ρ* of .46, the years 2016 and 2019 were weakly correlated with a *ρ* of .25, and the years 2017 and 2019 had a slightly stronger correlation with a *ρ* of .54 (Figure 3b). This reinforces the assessment that resistance to *S. sclerotiorum* is not strongly contingent on pathogen isolate, since CU11.19 and CU8.24 are unrelated (Derbyshire et al., 2019). The lower correlations when comparing either the 2017 or 2019 data sets with 2016 could be due to the environment in that year, which was particularly hot and dry. We noticed numerous plants that did not develop lesions even though they were clearly susceptible in subsequent assays.

**FIGURE 4:**
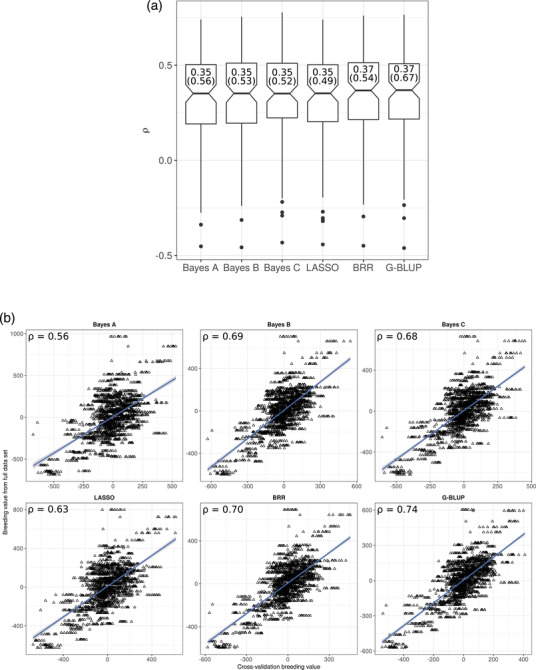
Cross‐validation assessment of different Bayesian priors using BGLR. (a) Median *ρ* (horizontal black bars) from 10 replicates of 11‐fold cross‐validation using five different Bayesian priors (*x* axis), compared with a genomic best linear unbiased predictor (G‐BLUP) linear mixed model. The boxes show second and third quartiles and whiskers show interquartile range. The numbers above horizontal black bars are predictive ability (at the top) and accuracy (below in brackets). Accuracy here is defined as predictive ability divided by *h* estimated from the model used. (b) The *x* axis shows genomic estimated breeding values (GEBVs) from all cross‐validations and the *y* axis shows corresponding GEBVs from the full model. The blue lines represent a least squares regression of *y* onto *x*. Pearson's *ρ* values are given in the top left. Overall, the G‐BLUP linear mixed and Bayesian ridge regression models (with a Gaussian prior) were the most accurate according to this assessment, highlighting the polygenic nature of the trait

We also assessed the impacts of different Bayesian priors on accuracy and predictive ability of genomic prediction models. Overall, the Bayesian models performed similarly to or worse than the genomic relationship matrix‐based additive G‐BLUP model (Figure 4). Predictive ability was .35 for the priors with heavier regularization, including Bayes A, B, and C, and the double‐exponential prior. The Bayesian ridge regression model with a Guassian prior had the highest predictive ability at .37, which was equal to the G‐BLUP model. Prediction accuracies for all Bayesian models ranged from about .49 to .56 (Figure 4a); this is considerably lower than that obtained for the G‐BLUP model (.67). Correlation between cross‐validation breeding values and breeding values estimated from the full data set was also lower (at .56–.7) for all of the Bayesian models than it was for the G‐BLUP model (.74). The best scoring prior for this measurement of prediction accuracy was the Gaussian prior at .7.

Using a Guassian prior in a Bayesian context is very similar to ridge regression or G‐BLUP. The other priors have much stronger regularization, which would likely accentuate this effect. The fact that the Gaussian prior was the most accurate through cross‐validation and that the G‐BLUP model was even better would suggest that the infinitesimal model is a good approximation for the genetic architecture behind sclerotinia stem rot resistance in a diverse population of *B. napus*. This is in line with some GWAS studies on this trait. For example, the most significant QTL markers identified by a study by Wu et al. (2016) explained about 5–6% of phenotypic variance each. Thus, this trait may be particularly suitable to genomic selection over selection based on a few QTL markers.

Overall predictive ability of about *ρ* = .35–.42, depending on model specification, for this trait appears moderate compared with some other canola traits such as seed oil content (*ρ* = .81) but is favorable compared with others such as seedling emergence (*ρ* = .29). It is similar, in fact, to the moderately heritable trait, lodging resistance, for which marker‐based estimates were correlated with phenotypes with *ρ* = .39 (Jan et al., 2016). However, our study is not directly comparable with these values for several reasons. First, we used a population of 218, whereas Jan et al. (2016) used a total population of 475 plants. Second, Jan et al. (2016) reported estimates of prediction accuracy of test‐cross performance, whereas we reported prediction accuracy for models fit on replicated data from inbred lines.

Our study may be more comparable to that of Werner et al. (2018), who reported genomic prediction accuracy on a diverse panel of 203 Chinese semi‐winter varieties tested over 2 yr for multiple traits. Some traits in this study were predicted with relatively high accuracy. For example, average cross‐validation *ρ* values for glucosinolate content and plant height were about .7 and .5, respectively. However, in one of the study years, oil content had a lower prediction accuracy of *ρ* ≈ .2. Ultimately, predictive abilities from models trained on diversity sets will be strongly affected by the precise linkage disequilibrium patterns and relationships between individuals within them. In more advanced stages of a breeding program, predictive ability and accuracy could be much higher, as populations are likely to be much less genetically diverse. For prebreeding variety selection, a model trained on a diversity set such as ours could, however, offer a more targeted approach over a large screen of random plants.

A limitation in our study is that we only tested 99 varieties in all three environments. Therefore, we could not assess prediction accuracy independently in each environment, which would be a better assessment of overall prediction accuracy for this trait. A model fit to more extensive data, incorporating multiple isolates of the pathogen across multiple environments, may be required for application. It may be particularly important to test disease resistance in a field context rather than in a pot trial as plants could respond differently to disease pressure in different conditions. This may be impractical for breeding since sclerotinia stem rot measurements are confounded in the field by varietal flowering time and strongly influenced by environmental conditions. If sclerotinia stem rot resistance breeding is limited to pot trials such as these, much work is needed to at least validate the best and worst scoring varieties with multiple pathogen isolates and, preferably, under multiple growth conditions. Inclusion of nonadditive genetic effects has the potential to improve prediction accuracy for this trait, which should be considered in any future application of genomic prediction to breeding canola for this trait.

## AUTHOR CONTRIBUTIONS

Yuphin Khentry: Investigation; Methodology; Writing‐review & editing. Anita Severn‐Ellis: Investigation; Methodology; Writing‐review & editing. Virginia Mwape: Investigation; Methodology; Writing‐review & editing. Nur Shuhadah Mohd Saad: Investigation; Methodology; Writing‐review & editing. Akeem Owolabi Taiwo: Investigation; Methodology; Writing‐review & editing. Roshan Regmi: Investigation; Methodology; Writing‐review & editing. Lone Buchwaldt: Resources. Matthew Denton‐Giles: Conceptualization; Investigation; Methodology; Writing‐review & editing

## CONFLICT OF INTEREST

The authors report no conflicts of interest.

## Supporting information



Supplemental Material

Supplemental Table S1. A list of *B. napus* varieties used in this study and their origins and descriptions.

Supplemental Table S2. All SNP genotypes used in this study, before filtering and imputation were performed.

Supplemental Table S3. 60K SNP data for the 105 winter accessions from the ASSYST population used for assessing population structure.
